# Quantitative Characterization of the Affected Zones in a Single Crystal Fe-6Si Steel Sheet by Fine Piercing

**DOI:** 10.3390/mi13040562

**Published:** 2022-03-31

**Authors:** Tatsuhiko Aizawa, Tomomi Shiratori, Tomoaki Yoshino, Yohei Suzuki, Kuniaki Dohda

**Affiliations:** 1 Surface Engineering Design Laboratory, SIT, Tokyo 144-0045, Japan; 2 Faculty of Engineering, University of Toyama, Toyama 930-8555, Japan; shira@eng.u-toyama.ac.jp; 3 Komatsu-Seiki Kosakusho, Co., Ltd., Suwa 392-0012, Japan; yoshino@komatsuseiki.co.jp (T.Y.); y-suzuki@komatsuseiki.co.jp (Y.S.); 4 Department of Mechanical Engineering, Northwestern University, Evanston, IL 60208, USA; dohda.kuni@northwestern.edu

**Keywords:** motor core, electrical steel sheet, single crystal, plastically affected zone by piercing, plastic straining, spinning, EBSD, crystallographic characterization

## Abstract

An iron loss in the motor core was often enhanced by formation of plastically affected zones in piercing the electrical steel sheets. A platform methodology to carry out quantitative evaluation of these affected zones in the pierced electrical steel sheets was proposed to search for the way to minimize the affected zone widths. A coarse-grained electrical steel sheet was employed as a work material for a fine piercing experiment under the narrowed clearance between the plasma-nitrided SKD11 punch and core-die. The shearing behavior by the applied loading for piercing was described by in situ measurement of the load-stroke relationship. The plastic straining in the single-crystal electrical steel sheet was characterized by SEM (scanning electron microscopy) and EBSD (electron back-scattering diffraction) to define the affected zone size and to analyze the rotation of crystallographic orientations by the induced plastic distortion during piercing. Integral and differentiation of spin rotation measured the affected zones. The effect of punch edge sharpness on these spin-rotation measures was also discussed using the nitrided and ion-milled SKD11 punch and core-die.

## 1. Introduction

A motor core is one of the most essential parts not only in electric vehicles but also in passenger and commodity cars [1]. In addition to a large-scaled driving motor-units, many small and tiny motors work in these automobiles to improve the comfort of driving conditions. Hence, the energy loss in working those motors must be reduced as far as possible to lower CO_2_ emission during driving [2]. As surveyed in [3], the energy inefficiency comes from various losses from defects in the shaped electrical steel sheets. In particular, the plastically affected zones left in the steel sheets by piercing is responsible for hysteresis loss [4]. Reduction of this affected zone size has become an engineering issue in fabrication of high-quality motor cores [5]. As analyzed in [6], this affected zone consists of the plastically and elastically affected zones and their mixture zones. This description of the affected zones was too empirical to discuss the effect of piercing or the punching-out process on the microstructure changes with iron loss.

In recent, the micromechanical analysis by EBSD (electron back-scattering diffraction) was highlighted to describe the local plastic flow of work materials [7,8,9]. KAM (kernel angle misorientation) was found to be in fairly good correlation to the equivalent plastic strains [10]. This EBSD analysis has another role, which is to describe the change of crystallographic orientations during the metal forming by using IPF (inverse pole figure) maps [11,12]. As proposed in [13], the induced distortion rate into a single-crystal electrical steel by piercing is composed of the elasto-plastic strain rate and the spin-rotation rate. This spin-rotation analyzed by EBSD provides a measure of the zones severely affected by piercing.

The present study concerns the quantitative characterization of the affected zones induced into a single crystal Fe-6Si by fine piercing. A coarse-grained, Fe-6Si grain-orientated electrical steel sheet with an average size of 10 mm was employed to select a single crystal with its specified crystallographic orientation for perforation by indentation of punch under the narrowed clearance. SEM (scanning electron microscopy)—EDX (electron diffraction X-ray spectroscopy) as well as EBSD were utilized to describe the shearing process when piercing by using the plasma-nitrided SKD11 punch and core-die. The pierced single-crystal specimen was prepared for EBSD analysis to describe the plastic straining and spin-rotation in fine piercing. The mechanically affected zone was defined by the KAM distribution and by the integral profile of spin rotations. The microstructure affected zone was also defined by the differentiation of spin-rotation. The effect of punch- and die-edge sharpness on these spin-rotation measures is also discussed by using the nitrided and ion-milled SKD11 punch and core-die.

## 2. Experimental Procedure

A single-crystal Fe-6Si was selected from the coarse-grained sheet as a test piece to analyze the plastic strains and spin rotation induced by the piercing process. The nitrided punch and core-die, and the CNC (computer numerical control) stamping system were utilized for this piercing experiment. SEM-EDX and EBSD were used for plastic strain and spin-rotation analyses.

### 2.1. Characterization of the Straining and Spinning in the Single-Crystal by Piercing

**Figure 1:**
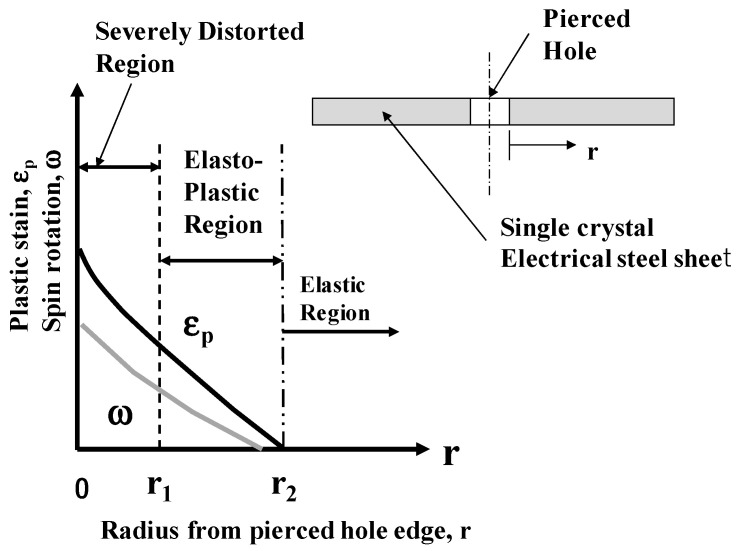
A platform model to characterize the piercing process into a single-crystal electrical steel sheet.

Each single crystal of electrical steel specimen had a thickness of 200 μm; it had a single crystal structure even in its thickness. The piecing process was identified as an axisymmetric indentation of rigid cylindrical punch into a single crystal work to simplify shearing in its depth. With the aid of the slip-line theory [14], a piercing test was identified as a one-dimensional model to quantitatively describe the distorted zones in the pierced crystal as depicted in Figure 1. Due to the axisymmetric mechanical condition, the spin rotations and the plastic strains related to the direction of depth were neglected; the radial and circumferential plastic strains (ε_r_ and ε_θ_) and the radial rotation (ω) were only effective to describe the material response in Figure 1.

As suggested in [15], when piercing under the narrow clearance, the severely distorted zone was limited to a vicinity of the pierced hole for r < r_1_. Since the initial crystallographic orientation of single crystal was different from the shearing direction, each zone in the single crystal was forced to significantly rotate itself together with plastic straining. This elasto-plastic zone was characterized as an area where the spin rotation (ω) and equivalent plastic strain (ε_p_) monotonously increased with decreasing from r = r_2_ to r = r_1_. These two zones were surrounded by the elastic zone. In the following EBSD analysis, ω and ε_p_, measure the affected zones.

### 2.2. Preparation of the Nitrided Punch and Core-Die

**Figure 2:**
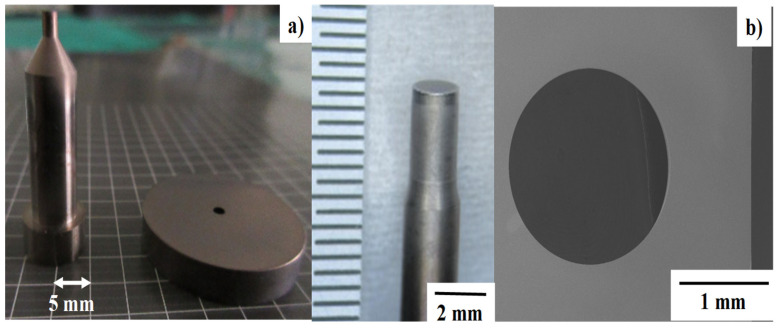
Two types of plasma-nitrided SKD11 punches. (**a**) As-nitrided SKD11 punch and die, and (**b**) nitrided and edge-sharpened SKD11 punch and die.

A pair of SKD11 punch and core-die was prepared and plasma-nitrided at 673 K for 14.4 ks by 70 Pa. Figure 2a shows the nitrided punch and die for a fine piercing experiment. The measured clearance between these punch and die is 1.5% of the sheet thickness. As shown in Figure 2b, the nitrided SKD11 punch with the diameter of 2.00 mm and core die were edge-sharpened by using the argon ion beam irradiation [16,17] to discuss the possibility to control the plastic straining and spin-rotation process in piercing.

### 2.3. Fine Piercing by CNC-Stamper

**Figure 3:**
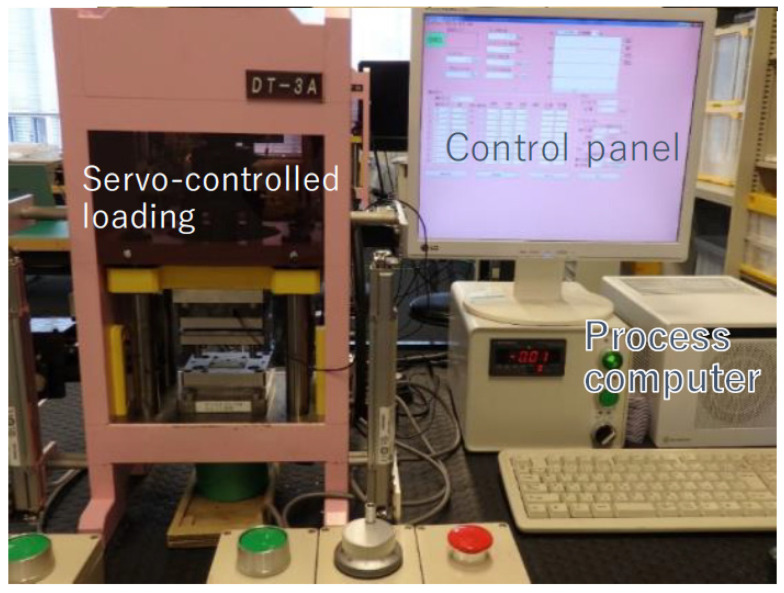
CNC-stamping system for a fine piercing experiment under nearly zero clearance between the nitrided punch and core-die.

Each punch and core-die pair was fixed into a cassette die set and cemented to upper and lower bolsters of the CNC (computer numerical control)-stamping system (Fine Metal Forming Laboratory, LLC.; Tokyo, Japan), as shown in Figure 3. A load-cell was embedded into the lower die to monitor the applied load.

### 2.4. Single-Crystal Fe-6Si Steel Work

**Figure 4:**
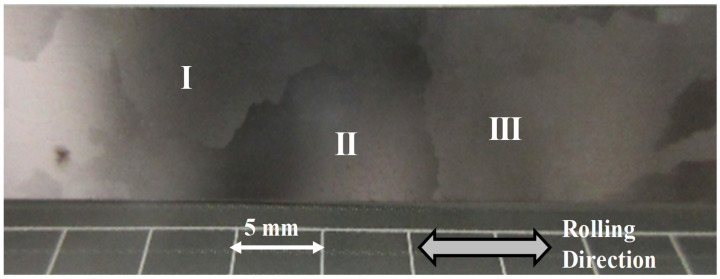
The coarse-grained Fe-6Si electrical steel sheet specimen with large single-grains I, II, and III aligned along the axis of easy magnetization <001> by rolling and annealing.

A grain-oriented electrical steel sheet was prepared by rolling and annealing processes as depicted in Figure 4. This coarse-grained sheet specimen had larger grains than 10 mm and smaller grains than 5 mm. Former grain orientations were often aligned along the axis of easy magnetization <001> by rolling and annealing; e.g., three large coarse grains I, II, and III had nearly the same crystallographic orientations, while latter grains had misorientation to this axis so that the effect of original crystallographic orientation on the affected zone formation by piercing was described by selecting these smaller grains. Each grain had homogeneous microstructure in the thickness of 0.2 mm.

### 2.5. Plastic Strain and Spin-Rotation Analysis by EBSD

The equivalent plastic strain was measured by the kernel misorientation angle in EBSD analysis. In particular, the high plastic strain zones or the slip-lines were identified as areas with high KAM. In parallel with plastic straining, each zone in the single crystal was distorted to change its crystallographic orientation. Hence, each orientation change induced a radial spin rotation from the original crystallographic orientation of single crystal as stated in [18]. Assuming that elastic spin rotation is negligibly small and the spin rotation is measured along the reference line from the reference point in elasticity in the pierced crystal, every zone locating from the reference point to the pierced hole edge gradually spin-rotated by itself. There are two ways to measure this spin-rotation in the pierced single crystal. One is an integration of the spin-rotation in each zone along the reference line from the reference point (t = 0) to the current point (t = s) by

(1) $$\sum \omega \;\left(s\right)=\int_{0}^{s}\omega {\left(t\right)}^{}\;\mathrm{dt}$$

where s is a coordinate along the reference line. Since each zone along the reference line is plastically strained in approaching to the pierced hole edge, this Σω (s) monotonously increases with s from zero to the length of reference line.

On the other hand, the spin-ration difference between neighboring zones, Δω (s), represents the differentiation of spin-rotation profile; e.g.,

(2) Δω(s)=ω(s)−ω(s−Δs)

When the neighboring two zones at s and Δs are only affected by gradual plastic straining, this Δω must be negligibly small or Δω ~ 0. If either zone at s is affected by the shear-straining in piercing process, Δω abruptly increases and decreases at the vicinity of s along the reference line.

## 3. Experimental Results

### 3.1. Piercing Process

**Figure 5:**
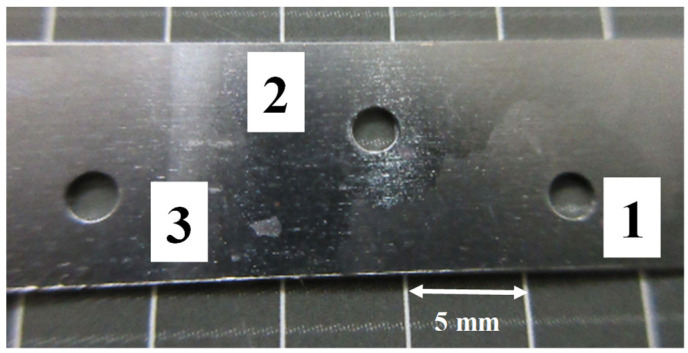
A pierced hole in each single crystal electrical steel sheets. Three holes were perforated into each crystal with different crystallographic orientation.

A pair of nitrided SKD11 punch and die in Figure 2a was utilized for the piercing process. Among several coarse grains in the specimen, three grains with n average size of 8 mm were selected as a work material in the following experiments. Figure 5 shows the perforated single Fe-6Si crystals by piercing experiments. Among several test pieces, the pierced crystal with No. 1 was selected for the following material characterization.

**Figure 6:**
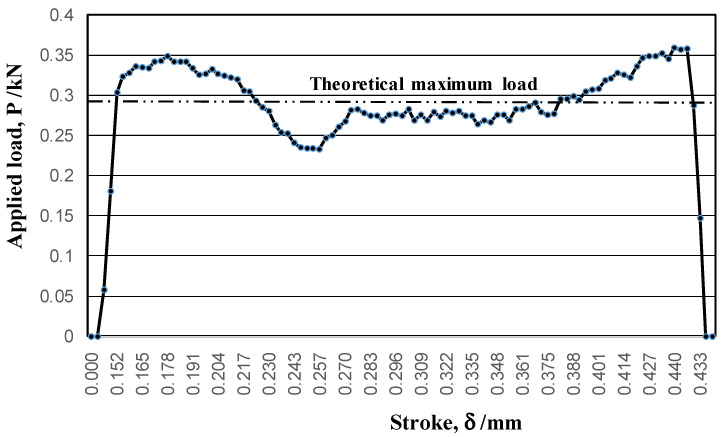
The applied load-stroke relationship in piercing a selected single-crystal electrical steel sheet.

The applied load was monitored by the load cell for each stroke. Figure 6 depicts the applied load-stroke relationship. Assuming that the piercing advances in simple shear stress, the maximum load (P_max_) was estimated to be P_max_ = πD × κ t, where D is the punch diameter, κ is the shear yield stress, and t is the sheet thickness. Since D = 2 mm, k = σ_y_/√3 MPa for the uniaxial yield stress, σ_y_ = 400 MPa, and t = 0.2 mm, P_max_ = 0.29 kN.

Except for the onset of indentation and the punching-out, the applied load slightly deviated from the theoretical maximum load; the piercing took place by simple shearing the Fe-6Si single crystal. At the onset of indentation, a multiaxial stress state was induced by shear-droop; the elasto-plastic separation also induced the multiaxial stresses when punching out the work.

**Figure 7:**
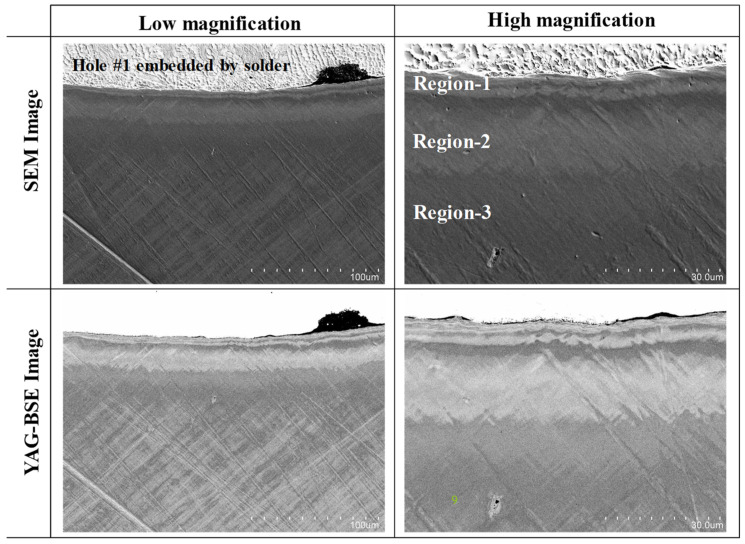
SEM analysis on the pierced single crystal electrical sheet.

Figure 7 depicts the SEM and YAG-BSE images on the vicinity of pierced hole in low and high magnifications. These images reveal that the single crystal near the pierced hole was divided into three regions; region-1 for 0 < r < r_1_ = 10 μm, region-2 for r_1_ < r < r_2_ = 30 μm, and region-3 for r > r_2_. Corresponding to Figure 1, the affected zones by piercing consisted of these three regions.

### 3.2. Analysis on the Plastic Strains by EBSD

**Figure 8:**
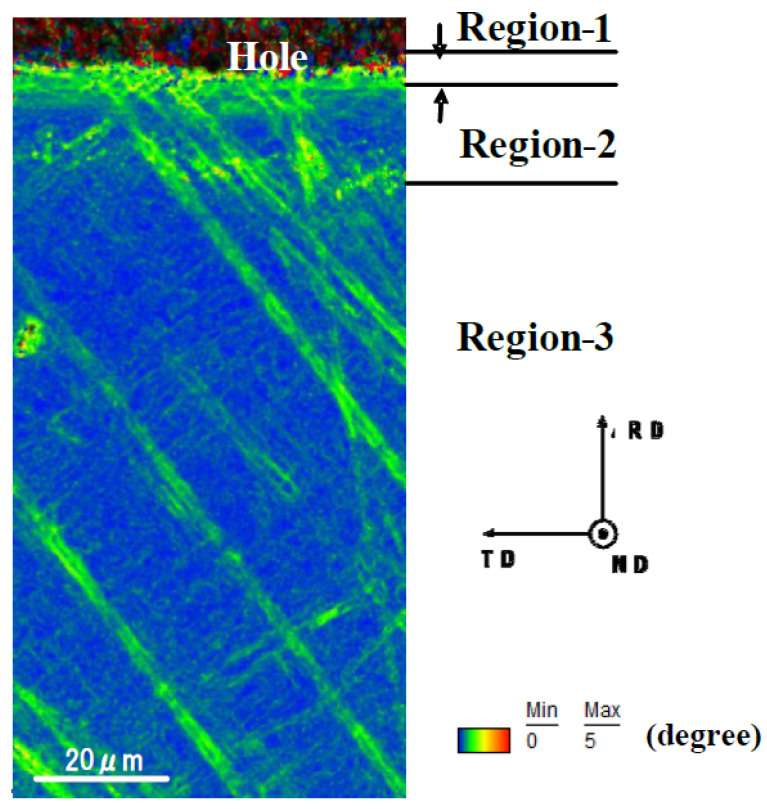
KAM distribution from the vicinity of pierced hole to the distanced area in the perforated single-crystal electrical steel sheet.

The plastic straining at the vicinity of pierced hole was described by using the KAM distribution as shown in Figure 8.

Expect for the noise by the mechanical polishing, nearly zero misorientation angle was induced in the region-3. This proves that region-3 was an elastic zone. Relatively higher strains were seen in the region-2, proving that the region-2 was an elasto-plastic zone. A significantly high misorientation angle was seen in the region-1. This suggests that this region-1 was severely strained by piercing.

### 3.3. Analysis on the Inverse Pole Figures by EBSD

**Figure 9:**
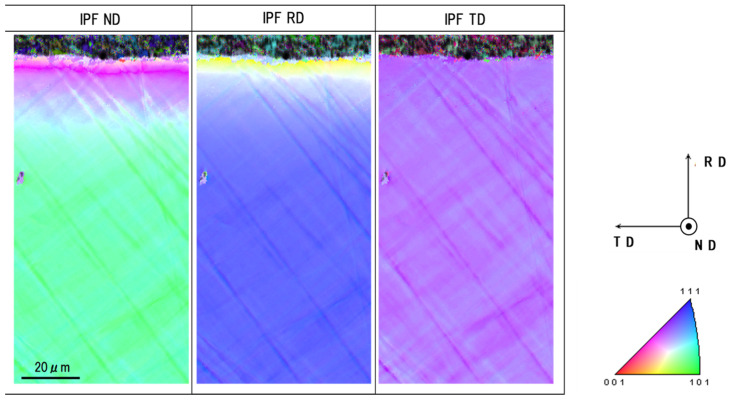
Inverse pole figure maps in the normal direction (ND), the rolling direction (RD), and the tangential direction (TD) on the pierced single-crystal electrical steel sheet.

The inverse pole figure mapping was utilized to describe the change in the crystallographic orientation on the pierced single-crystal surface as shown in Figure 9. On most of surfaces away from the pierced hole edge, no change was distinguished in the whole IPF mapping. These crystallographic orientations gradually changed in IPF maps in ND and RD when approaching to the vicinity of hole edge. In particular, a distinct orientation change was detected at a distance of 10 μm from the edge or at r = r_1_. On the other hand, the IPF map in TD was nearly the same as before piercing. This is because the axisymmetric plastic straining by piercing induced little crystallographic change in the circumferential direction.

We compared the microstructure change in Figure 7 with the crystallographic orientation change in Figure 9. The microstructure change of the region-2 in Figure 7 was just corresponding to the color-grating zone or the gradual crystallographic orientation in Figure 9. Each zone in the single crystal rotated itself by the plastic straining in region-2. In region-1, the microstructure in Figure 7 was characterized by two successive layers for 0 μm < r < 4 μm and 4 μm < r < 10 μm; each layer had two alternative microstructures. The former layer was not distinctly observed in Figure 9; the latter layer was distinguished as zones with the same crystallographic orientation. This comparison implies that the hole edge vicinity was first detected as the first layer and that the other kink in microstructure and its following zone with the same crystallographic orientation formed the second layer. That is, region-1 was composed of these two different layers.

### 3.4. Analysis on the Spin Rotation by EBSD

The spin rotation profile in the pierced single-crystal was measured from IPF to quantitatively describe the change in microstructure and crystallographic orientation in the above. Two measures were employed for this quantitative description; e.g., the integral and differentiation of spin rotation as defined by Equations (1) and (2), respectively.

**Figure 10:**
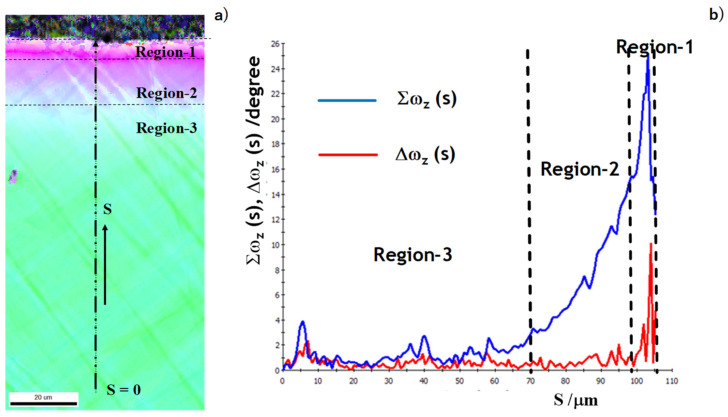
Differential and integral of the crystallographic spin rotation, ω_z_ in the normal direction, from the starting point (SP) to the end point (EP) on the pierced single-crystal electrical steel sheet. (**a**) A schematic view on the coordination s from SP and EP, and (**b**) differential and integral of ω_z_ along the axis from SP to EP.

As depicted in Figure 10a, these two measures were calculated as a function from the starting point (SP) to the end point (EP) along the profiling line. Since SP was selected sufficiently away from the pierced hole edge, this SP was free from the plastic straining by piercing. EP was selected at the nearest point to the hole edge. Figure 10b depicts the variation of two measures along the distance from SP to EP. In region-3, both measures were negligibly small; region-3 was free from plastic straining and stayed in the elastic state. In region-2, no significant increase was detected in Δω (s) for 70 μm < s < 100 μm; no significant change occurred in the crystallographic orientation. Σω (s) monotonously increased with s in this region; the crystallographic orientation gradually changed by the plastic straining in piercing the single crystal. In region-1, two peaks of Δω (s) were detected at s = 102 μm and 104 μm or r = 4 μm and 2 μm, respectively. This implies that the crystallographic orientation discontinuously changed at these two points. Two microstructure change lines in Figure 7 and Figure 9 were just corresponding to these two spikes observed in Δω profile.

The integral of spin rotation measured the continuous change from the elastic region to the elasto-plastic region during piercing the single-crystal work. The elastic state was measured by very few changes of integral; each zone in the elastic state had the same crystallographic orientation as the mother single crystal without any plastic strains. The continuous plastic straining and crystallographic change in the elasto-plastic state was measured by the monotonous increase of Σω (s).

The differentiation of spin rotation was completely insensitive to the elastic state in region-3 and the elasto-plastic state in region-2. The discontinuous change of crystallographic orientation was only measured by the significant peak in Δω. The microstructure-affected region-1 was characterized by this measure.

### 3.5. Analysis on the Effect of Punch Edge Sharpness on the Spin Rotation Profiles

**Figure 11:**
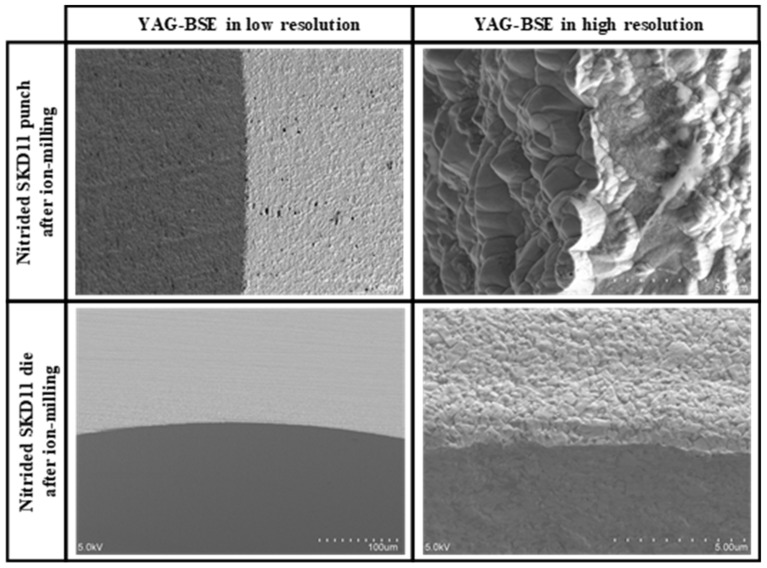
YAG-BSE image on the nitrided SKD11 punch and die after ion-milling for 14.4 ks both in low and high resolutions.

**Figure 12:**
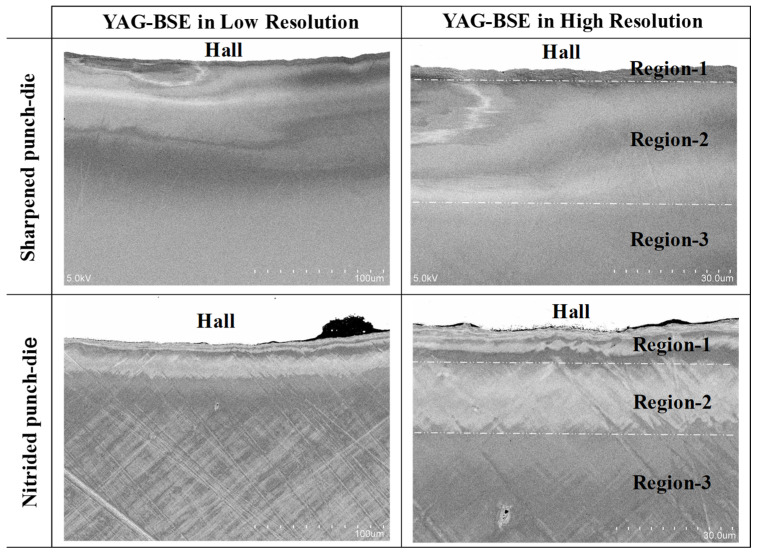
Comparison of YAG-BSE images in low and high resolutions on the pierced single crystal near the hole edge by using the sharped and normal punch—die systems.

The elasto-plastic regions and microstructural change induced by piercing were influenced by the punch and die edge sharpness. Figure 11 shows the SEM images of nitrided SKD11 punch and die after argon ion-milling for 14.4 ks or 4 h. Although the surface roughness was left on the punch head and side surfaces, the punch edge was sharped to have an edge width around 1 μm. The die edge was also sharpened in a similar manner to the punch edge. In a similar manner to the piercing experiment in Section 3.4, these edge-sharpened punch and die were utilized in the piercing experiment. Figure 12 compares the YAG-BSE images in low and high resolutions on the pierced single crystal near the hole edge by using the sharped and normal punch—die systems.

**Figure 13:**
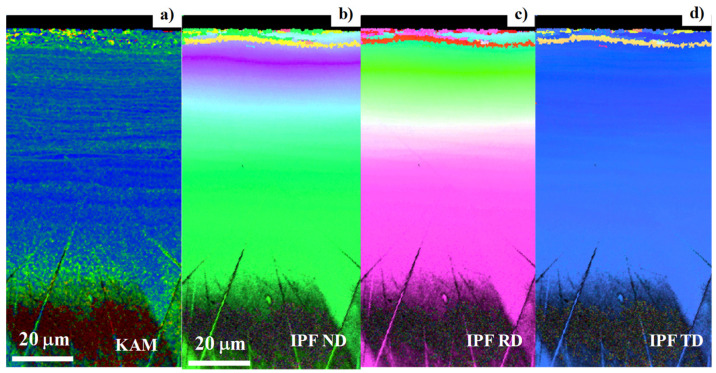
KAM and IPF mappings on the pierced single crystal near the hole edge by using the sharpened punch–die system. (**a**) KAM distribution, (**b**) IPF in ND, (**c**) IPF in RD, and (**d**) IPD in TD.

When using the sharpened punch and die system, the affected zone by piercing process was also characterized three regions. Due to the edge sharpness, the width of region-1 was reduced from 10 μm to 5 μm. The width of region-2 became nearly the same irrespective of the edge sharpness. Figure 13 depicts the KAM distribution and IPF mapping at the vicinity of the pierced hole. The high KAM zones were formed in the circumferential direction; the high KAM zone density increased from region-2 to the region-1. In correspondence to this plastic straining, IPF mapping gradually changed itself as the color grading in Figure 13b–d. That is, the crystallographic orientation changed by spin rotation in each zone of region-2 together with plastic straining toward region-1. In region-1, two lines with distinct change in crystallographic orientations were detected in Figure 13b–d.

**Figure 14:**
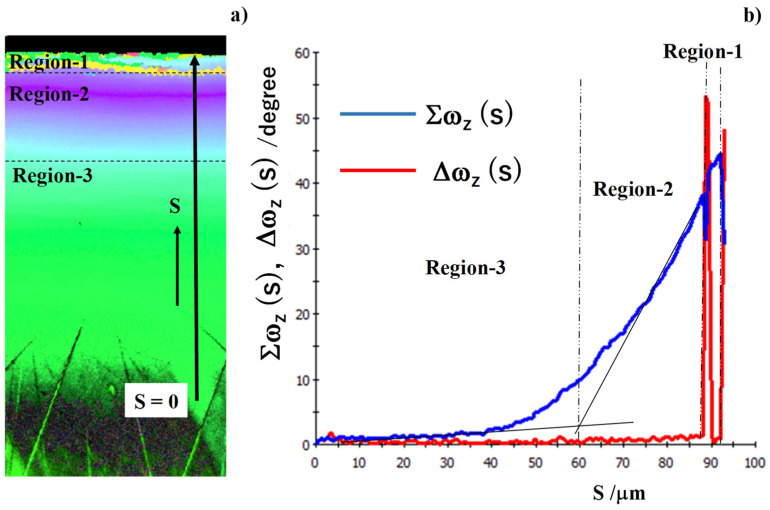
Differential and integral of the crystallographic spin rotation, ω_z_ in the normal direction, from the starting point (SP) to the end point (EP) on the pierced single-crystal electrical steel sheet. (**a**) A schematic view on the coordination of SP and EP, and (**b**) differential and integral of ω_z_ along the axis from SP to EP.

In a similar manner to Figure 11, we analyzed the three regions by using two measures in spin rotation. Both the integral and differentiation of ω (s) were calculated along the profiling line in Figure 14a. Region-3 was characterized by Σω ~ 0 and Δω ~ 0. The crystallographic orientation change was negligibly small; region-3 remained elastic. Region-2 was characterized by monotonously increasing Σω and nearly zero Δω. Each zone in region-2 was elasto-plastically strained to gradually change its crystallographic orientation from the original orientation to the shearing direction. Region-1 in the vicinity of the pierced hole was characterized by two spikes in Δω (s) and two drops in Σω (s). These discontinuous changes were directly induced into Δω and Σω by piercing process. The first discontinuous spike on the border between the region-2 and region-1 was caused by the curvature change in the bending distortion during piercing. The second discontinuous spike was just a cutting edge by shearing the hole.

Comparing Figure 10 and Figure 14, the width of region-2 was nearly the same, but the width of region-1 was reduced by using the edge-sharpened punch and core-die for piercing. That is, the contribution of bending distortion to affected zone formation was reduced by piercing the electrical steel sheet with the use of edge-sharpened punch and core-die. The applied stress concentrated at the vicinity of the punch edge in shearing the steel sheet in a similar manner to the rigid punch indentation with formation of slip bands in the plastically deforming work [14].

## 4. Discussion

The affected zones have been discussed by microstructural analysis on the disordered magnetic zones by piercing the polycrystalline electrical steel sheets [4,6,19] or by the hardness profile measurement on the cross-section of the pierced polycrystalline electrical steel sheets [20]. Those studies are enough to describe the relationship between the microstructure change and the iron loss, but have no means to investigate the effect of the piercing process on the crystallographic changes in the electrical steel sheet. The present experimental procedure with the use of coarse-grained electrical steel sheets enabled us to describe the plastic straining and crystallographic spin-rotation in piercing the selected single-crystal electrical steel.

KAM, GAM (grain average misorientation), and GOS (grain orientation spread) were first utilized to describe the local plastic straining [21,22,23]. As stated in [21], these measures had good correlation to the plastic stains up to 20%. The fatigue damage was also analyzed by KAM to describe the relationship between dislocation structures and damages in [22]. Furthermore, the punched dual stainless steels were also analyzed by KAM to find a good correlation between the KAM and the pre-strain [23]. As seen in Figure 8 and Figure 13, the plastic strains advanced radially during the piercing; the radial plastic strains were induced by piercing. In particular, region-1 was much strained at the vicinity of the pierced hole.

The fine piercing process into single crystal Fe-6Si alloys was described by SEM and EBSD to define the affected zones induced by piercing. These affected zones were categorized into three regions. Region-1 was a severely affected zone with the crystallographic orientation change. Region-2 was an elasto-plastically strained zone with the gradual crystallographic orientation change from the intrinsic orientation to each single-crystal. Region-3 was an elastically strained zone, surrounding these two zones, without significant crystallographic change. KAM measured the difference between region-2 and -3 but did not describe each region quantitatively. As seen in Figure 8 and Figure 13, the plastic straining accompanied the crystallographic change in the ND and RD. The plastic distortion rate tensor was composed of the plastic strain rate tensor and the crystallographic spin-rotation rate tensor, following [24,25]. In the polycrystalline materials, the induced spin-rotation rate tensor was constrained by the grain boundary; this sensor in each grain was difficult to be used as a measure to evaluate the damage distribution through the materials by punching as stated in [18]. In the case of single crystal work material, its constituent zone was spin-rotated by the asymmetric part of plastic distortion rate tensor. The profiling of spin-rotation in each zone provided the crystallographic change between the adjacent zones. In particular, when punching a thin single crystal by the cylindrical punch, a plastic distortion in the radial direction was induced as seen in Figure 9 and Figure 13. The profiling of this spin rotation in the radial direction provided a measure to quantitatively evaluate the crystallographic evolution during the piercing.

The integral and differentiation of spin rotation, or Σω and Δω, were defined to quantitatively analyze these affected zones. Region-1 was characterized by two discontinuous crystallographic orientation changes. Two spiky peaks were observed in the Δω profile; the first peak represented the large spin rotation by shearing process and the second peak was induced by strain concentration during the local bending. As seen in Figure 9 and Figure 13, the IFP maps both in ND and RD significantly changed by themselves at these spiky peaks in Δω. Region-2 was featured by the monotonous change in the crystallographic orientation. Although Δω was negligibly small or very slightly increased, Σω increased in linear or slightly in nonlinear fashion along the profiling lime. This monotonous behavior of Δω and Σω revealed that each zone in the single-crystal was elasto-plastically strained to change its crystallographic orientation toward the slipping direction for bending. Region-3 was redefined by nearly zero Δω and negligibly small Σω, where the crystallographic orientation deviated in each zone by its elastic straining.

Through measuring these Σω and Δω profiles, two types of affected zones were formed by piercing the single crystal electrical steel sheets. The primary affected zone directly by the shearing process was characterized by significant crystallographic change. Most of domains near the pierced hole were forced to rotate to the shearing direction. The secondary affected zone by the piercing process was characterized by the gradual crystallographic change. Each spin rotation increased up to the criticality where the curvature is applied by local bending [26]. This formation of two affected zones was dependent on the punch edge sharpness.

We considered the edge-sharpness effect on the formation of affected zones by piercing. Region-2 and region-3 were insensitive to the punch and die edge sharpness. The region-2 width reached 30 μm when using the mechanically ground and nitrided SKD11 punch, and it slightly reduced to 25 μm when using the iron milled and nitrided SKD11 punch. The region-1 width was much reduced from 10 μm to 5 μm by using the edge-sharpened punch. This reveals that the edge-sharpness influenced the work material distortion during the piercing process. In general, as stated in [27], the simple shearing of the work sheet accompanied the local bending distortion within the clearance between punch and die. Remember that two spiky peaks of Δω in Figure 10 and Figure 14 represented the maximum curvature of work sheet by local bending and the simple shearing, respectively. Reduction of the reguon-1 width indicated that local bending and simple shearing occurred in a shorter distance.

Among the three regions in the affected zone of work sheet by piercing, the discontinuous crystallographic change in region-1 was responsible for deterioration of the magnetic zones. Since the crystallographic orientation was continuously spin-rotated by local bending in region-2, several zones neighboring region-1 were damaged in magnetism. The edge sharpness in the punch and die was effective to reduce the magnetic zone damages by reducing the region-1 width. In addition to this edge sharpening, the laser-trimmed punch edge and side-surface had a possibility to further reduce the magnetic distortion by minimizing the local bending effect. Following [28], the homogeneously sharp edge profile and the nano-structured side surface profile in the piercing punch provided a way to generate the fully burnished and metallic shining hole surface without the fractures. This is because the local bending effect was significantly suppressed by the stress concentration at the vicinity of sharp edge even under the relatively wide clearance.

The grain-oriented and non-oriented electrical steel sheets have been selectively utilized in each industrial application [29,30]. The effect of grain orientation distribution on the plastic anisotropy of electrical steel sheets must be taken into account to reduce the affected damages by the punching process [31,32,33,34]. The present method has the potential to experimentally evaluate this effect by selecting the coarse grains with different crystallographic orientations for fine piercing and by analyzing the KAM, the IPF, and the spin rotation profile through the precise EBSD analysis.

## 5. Conclusions

The affected zones in a single crystal Fe-6Si sheet by piercing were quantitatively analyzed by SEM and EBSD. They were categorized into three regions. The region away from the pierced hole was only elastically strained where its constituent zone had nearly the same crystallographic orientation as the original single crystal. The region near the pierced hole was elasto-plastically strained where the crystallographic orientation continuously rotated from the original orientation to the shearing direction by the local bending. The region at the vicinity of hole was severely damaged where the crystallographic orientation changed discontinuously. Both the integral and differentiation of spin rotation, or Σω (s) and Δω (s), quantitatively measured each categorized region in the affected zones. In the elastic region, Σω (s) ~ 0 and Δω (s) ~ 0. In the elasto-plastic region, Σω (s) monotonously increased with Δω (s) ~ 0. Since the gradient of Σω (s) was nearly constant, the crystallographic orientation was increased by constant curvature. In the damaged region, two spiky peaks were observed in Δω (s); the crystallographic orientation discontinuously changed at two points. In particular, the border between the elasto-plastically strained and damaged regions was characterized by snap-through from the continuous crystallographic orientation change by local bending to the declined orientation on the shear droop at the vicinity of pierced hole.

The present method is effective to describe the edge sharpness effect on the piercing behavior. The piercing process by the mechanically ground and nitrided SKD11 punch is characterized by the dull increase of Σω (s) in the elasto-plastically strained region and by the two low intensity peaks in Δω (s) in the damaged region. On the other hand, when using the edge-sharped punch, its affected regions by piercing are measured by the steep, monotonous increase of Σω (s) and two high intensity peaks in Δω (s). This difference comes from the mechanical condition where the simple shearing state prevails in the piercing process and the local bending state is more limited near the vicinity of the pierced hole when using the edge-sharpened punch and die.
